# Anti-CD45RC antibody immunotherapy prevents and treats experimental autoimmune polyendocrinopathy–candidiasis–ectodermal dystrophy syndrome

**DOI:** 10.1172/JCI156507

**Published:** 2022-04-01

**Authors:** Marine Besnard, Céline Sérazin, Jason Ossart, Anne Moreau, Nadège Vimond, Léa Flippe, Hanna Sein, Grace A. Smith, Stefania Pittaluga, Elise M.N. Ferré, Claire Usal, Ignacio Anegon, Annamari Ranki, Michail S. Lionakis, Pärt Peterson, Carole Guillonneau

**Affiliations:** 1 Université de Nantes, Inserm, Centre de Recherche en Transplantation et Immunologie, UMR 1064, Institut de Transplantation Urologie-Néphrologie, Nantes, France.; 2 Anatomie et Cytologie Pathologiques, Centre Hospitalier Universitaire Nantes, Nantes, France.; 3 AbolerIS Pharma, Nantes, France.; 4 Molecular Pathology, Institute of Biomedicine and Translational Medicine, University of Tartu, Tartu, Estonia.; 5 Laboratory of Pathology, Center for Cancer Research, National Cancer Institute, Bethesda, Maryland, USA.; 6 Fungal Pathogenesis Section, Laboratory of Clinical Immunology and Microbiology, National Institute of Allergy and Infectious Diseases, National Institutes of Health, Bethesda, Maryland, USA.; 7 Department of Dermatology and Allergology, Clinicum, University of Helsinki and Helsinki University Hospital, Helsinki, Finland.

**Keywords:** Autoimmunity, Therapeutics, Autoimmune diseases, Immunotherapy, Tolerance

## Abstract

Targeted monoclonal antibody (mAb) therapies show great promise for the treatment of transplant rejection and autoimmune diseases by inducing more specific immunomodulatory effects than broadly immunosuppressive drugs routinely used. We recently described the therapeutic advantage of targeting CD45RC, expressed at high levels by conventional T (Tconv) cells (CD45RC^hi^), their precursors, and terminally differentiated T (TEMRA) cells, but not by regulatory T cells (Tregs; CD45RC^lo/–^). We demonstrated efficacy of anti-CD45RC mAb treatment in transplantation, but its potential has not been examined in autoimmune diseases. Autoimmune polyendocrinopathy–candidiasis–ectodermal dystrophy (APECED) is a rare genetic syndrome caused by loss-of-function mutations of autoimmune regulator (AIRE), a key central tolerance mediator, leading to abnormal autoreactive T cell responses and autoantibody production. Herein, we show that, in a rat model of APECED syndrome, anti-CD45RC mAb was effective for both prevention and treatment of autoimmune manifestations and inhibited autoantibody development. Anti-CD45RC mAb intervention depleted CD45RC^hi^ T cells, inhibited CD45RC^hi^ B cells, and restored the Treg/Tconv cell ratio and the altered Treg transcriptomic profile. In APECED patients, CD45RC was significantly increased in peripheral blood T cells, and lesioned organs from APECED patients were infiltrated by CD45RC^hi^ cells. Our observations highlight the potential role for CD45RC^hi^ cells in the pathogenesis of experimental and human APECED syndrome and the potential of anti-CD45RC antibody treatment.

## Graphical abstract

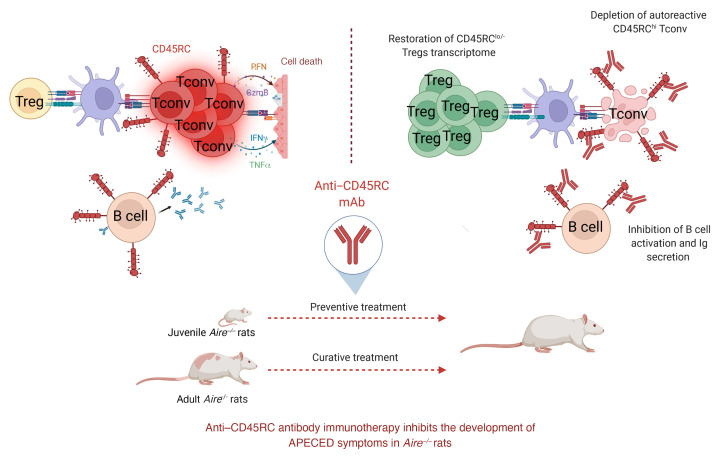

## Introduction

Disruption of central tolerance inherently triggers the development of autoimmune diseases with severe or life-threatening symptoms. The autoimmune polyendocrinopathy–candidiasis–ectodermal dystrophy (APECED) syndrome, also known as autoimmune polyglandular syndrome type 1 (APS-1), is one of these diseases (1). This rare inherited disease (more common among certain populations, such as Finns [1:25,000] and Sardinians [1:14,000]) is caused by a partially defective thymic function due to the loss of autoimmune regulator (AIRE), a critical gene for the negative selection and development of thymocytes. In physiological conditions, AIRE allows the expression of thousands of tissue-restricted antigens, i.e., self-antigens, in the thymus. These are presented to developing thymocytes, and those that recognize self-antigens undergo clonal deletion or deviation into the regulatory T cell (Treg) lineage (2). In APECED patients, AIRE function is impaired, the T cell repertoire is skewed, and autoreactive conventional T (Tconv) cells escape deletion while Tregs’ function is altered (3), inducing several life-threatening autoimmune manifestations (2, 4, 5).

APECED patients harbor an average of 5 to 20 manifestations, including chronic mucocutaneous candidiasis, hypoparathyroidism, adrenal insufficiency (Addison disease), alopecia, vitiligo, exocrine pancreatic failure, and autoimmune hepatitis (5–8). Most of the affected organs display strong lymphocytic infiltration and organ lesions that associate with the presence of tissue-specific autoantibodies (9). Indeed, the production of a wide autoantibody repertoire, and especially anti-cytokine autoantibodies, is characteristic of this syndrome (10).

APECED patients currently receive purely symptomatic treatment to control their clinical disease and symptoms: hormone replacement therapy, antifungal drugs, and immunosuppressive medications (6, 11, 12). Rituximab or any other conventional immunosuppressive drug has not proven efficient in preventing APECED-associated organ destruction, although a combination of azathioprine or mycophenolate mofetil and rituximab was recently reported to be beneficial in APECED pneumonitis patients (13). The long-term use of immunosuppressive drugs increases the risk of developing complications, such as cancer and/or infection (2, 14), a serious concern for APECED patients, who are already prone to chronic mucosal *Candida* infection (15). The life expectancy of APECED patients is reduced and has not increased in recent decades (16). Thus, identification of targeted therapeutic interventions is needed to improve the morbidity and mortality associated with this syndrome.

Despite the insights gained from these mouse models into the etiology and pathology of APECED, substantial phenotypic and clinical differences exist between AIRE-deficient mice and human APECED patients (17). Several years ago, we developed a model of *Aire^–/–^* rats that ultimately exhibit manifestations resembling the ones seen in APECED patients, including nephritis, pneumonitis, and pancreatic failure (17–19). They also harbor visible manifestations, such as alopecia, vitiligo, and nail dystrophy, which could be very useful for facile clinical follow-up during preclinical studies. In addition, and in agreement, *Aire^–/–^* rats produce many autoantibodies, including against cytokines, similarly to APECED patients (18).

CD45RC is the only isoform of the CD45 molecule to be highly expressed by CD4^+^ and CD8^+^ T cells exhibiting a Th1 proinflammatory profile, as well as naive precursors of Th1 cells and terminally differentiated T (TEMRA) cells, and at the same time, not expressed or very weakly expressed by Th2 cells and Tregs (20–22). We recently showed that monoclonal antibody (mAb) against CD45RC can induce tolerance in heart transplantation in rats and immune-humanized immunodeficient NSG mice with transplanted human skin (23); in graft-versus-host disease in mice, rats, and NSG mice (24); and in a rat model of Duchenne’s muscular dystrophy, another rare genetic disease with inflammatory components (25). We have previously shown that anti–rat CD45RC mAb acts by depleting CD45RC^hi^ Tconv cells in vitro and in vivo while preserving and potentiating CD45RC^lo/–^ Tregs in vivo, thus rebalancing the Treg/Tconv balance (23, 24). Since both Tconv cells and Tregs occupy a central position in APECED, we reasoned that anti-CD45RC mAb administration would be able to improve signs of autoimmunity in APECED syndrome by restoring the functional balance between Tconv cells and Tregs and the immune equilibrium.

We thus assessed the potential of anti-CD45RC mAb administration to prevent and/or treat APECED using the *Aire^–/–^* rat model. We show that anti-CD45RC mAb administration markedly decreased the number and severity of APECED lesions both when used as prevention and as treatment. We demonstrate that this effect was mediated by the specific depletion of CD45RC^hi^ Tconv cells with preservation/restoration of CD45RC^lo/–^ Tregs and inhibition of CD45RC^hi^ B cells. We also show that, in vivo, anti-CD45RC mAb administration reduced the production of tissue-specific and anti-cytokine autoantibodies. Finally, we show that CD45RC expression was dysregulated and significantly enhanced in CD4^+^ and CD8^+^ Tconv cells among peripheral blood T cells from APECED patients and that CD45RC^+^ cells infiltrated autoimmunity-affected tissues from APECED patients. Altogether, these results support a pathogenic role for CD45RC^hi^ cells in APECED and demonstrate that anti-CD45RC mAb immunotherapy may offer an effective therapeutic approach for use in patients with APECED syndrome.

## Results

### Preventive administration of anti-CD45RC mAb in juvenile Aire^–/–^ rats inhibits the development of visible autoimmune pathology.

**Figure 1:**
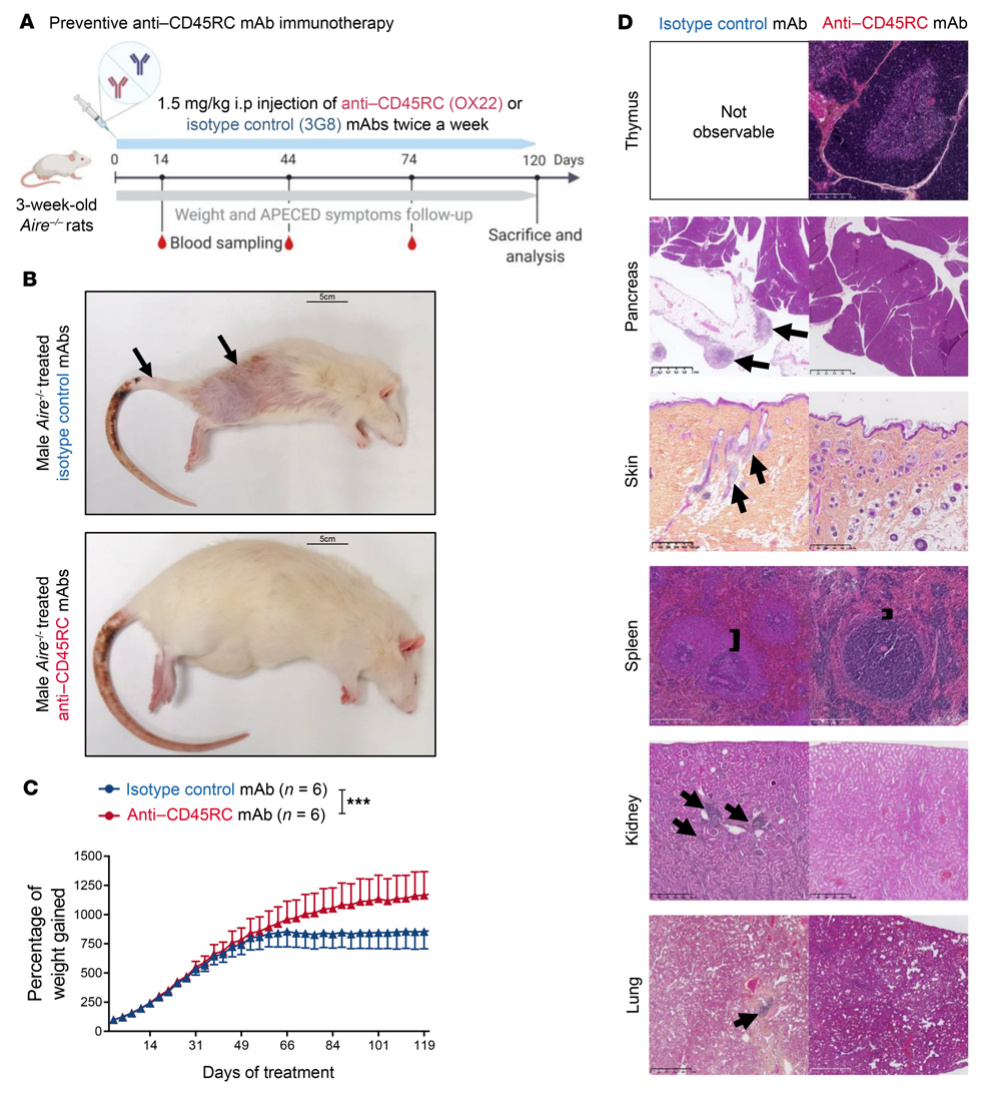
Anti-CD45RC mAb treatment controls the development of autoimmune lesions in *Aire^–/–^* rats. (**A**) Schematic of the protocol of administration of isotype control or anti-CD45RC mAbs as prevention in 3-week-old *Aire^–/–^* rats. (**B**) Representative photographs of *Aire^–/–^* rats after 4 months of treatment with either isotype control (*n =* 13) or anti-CD45RC mAbs (*n =* 13). Arrows indicate the alopecia- and vitiligo-like manifestations. Scale bars: 5 cm. (**C**) Evolution of weight gain in *Aire^–/–^* male rats during treatment with isotype control (*n =* 6) or anti-CD45RC mAbs (*n =* 6) until sacrifice. ANOVA comparing curves: ***P < 0.001. (**D**) Representative pictures of H&E histological analysis of organs from *Aire^–/–^* rats at the end of the 4-month treatment with isotype control (*n =* 7) or anti-CD45RC mAbs (*n =* 7). Black arrows indicate lesions and mononuclear cell infiltrates. Black bars indicate the marginal zone. Scale bars: 250 nm (thymus and spleen), 500 μm (skin and kidney), and 1 mm (pancreas and lung).

We first established a protocol of treatment to assess the in vivo effects of preventive anti-CD45RC immunotherapy. We began treatment of *Aire^–/–^* rats just after weaning at 3 weeks of age, prior to any visible pathology (weight loss, skin discoloration, and fur loss), and continued treatment for 4 months with 2 injections per week of either the anti-CD45RC or the isotype control mAb (Figure 1A). None of the animals had been sacrificed before the end of the experimental setup. At the end of the 4 months of treatment, all rats from the isotype control group (*n =* 13, 6 independent experiments) showed visible manifestations of APECED, i.e., alopecia and vitiligo, which manifests as patchy hair loss throughout the body, and skin depigmentation visible at the base of the tail in particular (Figure 1B, top, and Table 1). By contrast, in the group of *Aire^–/–^* rats treated with the anti-CD45RC mAb (*n =* 13, 6 independent experiments), none of the animals showed any of the aforementioned visible signs of APECED (Figure 1B, bottom, and Table 1). Analysis of the percentage of weight gained during the treatment further revealed that *Aire^–/–^* rats treated with the isotype control abruptly stopped gaining weight after only 2 months of treatment, whereas anti-CD45RC–treated *Aire^–/–^* rats slowly reached a plateau but continued their growth until the end of the experiment, and at the end of the treatment period the difference in weight between the 2 groups was 32% (Figure 1C). These visible observations were consistent with the histological analysis of the organs (Figure 1D, left). At 5 months of age, all *Aire^–/–^* rats treated with the isotype control mAb showed autoimmune organ lesions consistent with what was previously described (Table 1 and ref. 18). Briefly, we observed lesions of the pancreas with small zones of lymphocytic infiltration, with 25% of the animals developing very severe pancreatitis with large lymphocytic infiltrates, fibrosis, and acinar metaplasia. Skin tissues showed reduced numbers of hair follicles and fragmented hairs. The marginal zone of the spleen follicles was abnormally large, indicating an exaggerated immune stimulation. Kidneys and lungs presented lymphocytic infiltrations, as previously demonstrated in untreated *Aire^–/–^* rats (18). Thymi from *Aire^–/–^* rats treated with the isotype control mAb could not be detected. In contrast, after anti-CD45RC mAb immunotherapy, all *Aire^–/–^* rat thymi were present with preserved architecture, and the cortex and medulla were easily distinguishable on H&E staining. In addition, after anti-CD45RC mAb immunotherapy, histological analysis of pancreas, skin, kidneys, and lungs showed no lymphocytic infiltration, and the marginal zone of splenic follicles was normalized (Figure 1D, right). Taken together, these findings demonstrated that preventive anti-CD45RC immunotherapy given as sole agent protects from the appearance of autoimmune organ lesions in the setting of AIRE deficiency in juvenile rats.

### Preventive administration of anti-CD45RC mAb decreases autoantibody production.

**Figure 2:**
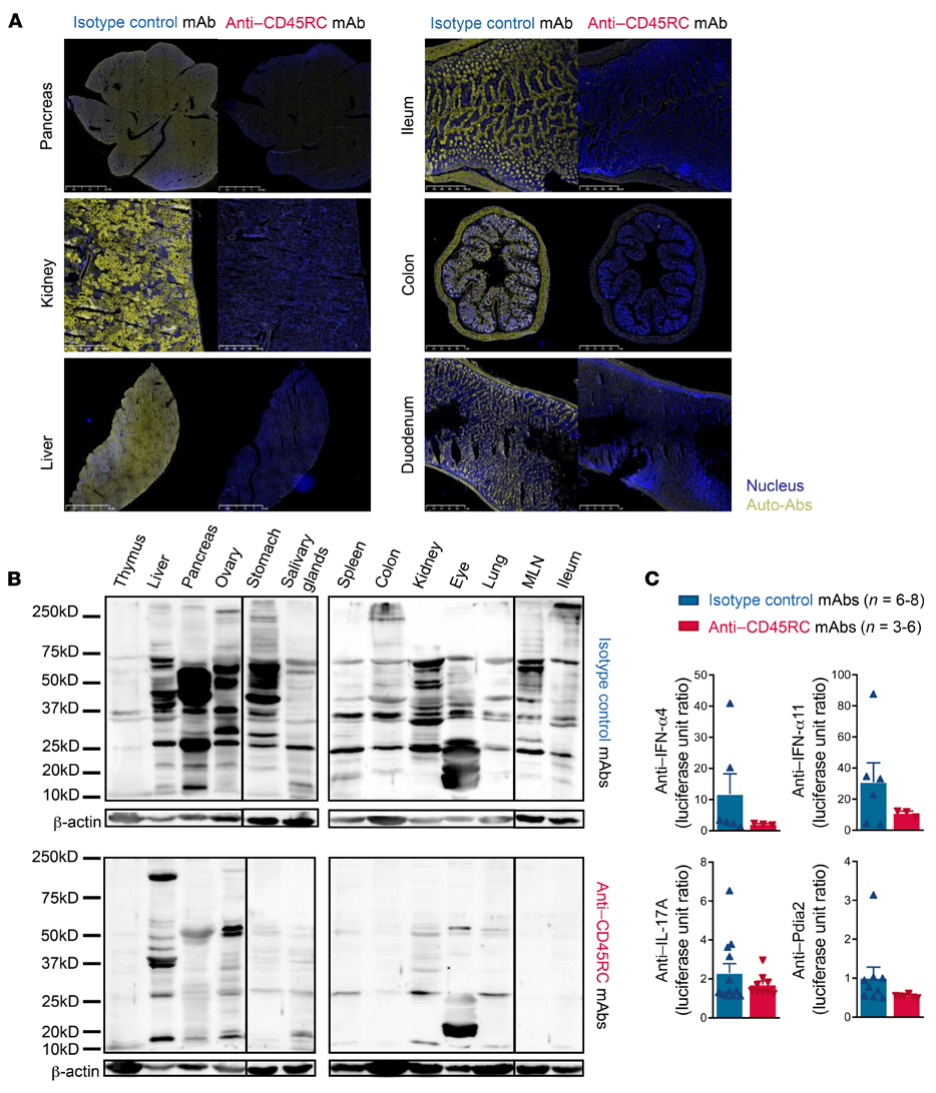
Preventive anti-CD45RC mAb immunotherapy decreases the production of autoantibodies in *Aire^–/–^* rats. (**A**) Tissue sections of organs from *IgM^–/–^* rats were incubated with sera of *Aire^–/–^* animals at 4 months of treatment with anti-CD45RC or isotype control mAb. Autoantibodies are depicted in yellow and DAPI in blue. Original magnification, ×20. Images are representative of 3 different experiments. (**B**) Sera from anti-CD45RC or isotype control mAb–treated *Aire^–/–^* rats at 4 months of treatment were incubated on Western blot membranes after migration and transfer of tissue-specific self-antigens from *IgM^–/–^* rats. Binding of autoantibodies was revealed using an anti-rat IgG biotin-coupled antibody and avidin peroxidase. β-Actin was used as a loading control. Data are representative of 5 different experiments. MLN, mesenteric lymph nodes. (**C**) Anti-cytokine and tissue-specific autoantibodies were quantified by LIPS assay using sera from anti-CD45RC or isotype control mAb–treated *Aire^–/–^* rats at 4 months of treatment. Normalization was achieved by division of the obtained value by the mean values obtained from WT animals. Mann-Whitney analysis showed no significant difference between the 2 groups.

Autoantibodies are a major feature of APECED syndrome. Thus, we first measured the level of circulating antibodies in the sera of *Aire^–/–^* animals treated with the anti-CD45RC or the isotype control mAb (Supplemental Figure 1A; supplemental material available online with this article; https://doi.org/10.1172/JCI156507DS1). None of the different immunoglobulin subclasses had levels significantly changed in the serum of *Aire^–/–^* rats treated with the anti-CD45RC or the isotype control mAb compared with WT animals. To detect tissue-specific autoantibodies directed against tissues, we analyzed the binding of serum IgG from isotype or anti-CD45RC mAb–treated rats to different tissues from B cell/immunoglobulin–deficient rats, which were used to avoid nonspecific staining. We previously showed that no reactivity could be observed with *Aire^+/+^* sera (18). Microscopy analysis of immunostaining of sera from isotype control mAb–treated *Aire^–/–^* animals on several tissues showed high reactivity (Figure 2A, left), consistent with the presence of autoantibodies in sera from untreated *Aire^–/–^* animals, as shown before (18). In contrast, we observed very low staining or no staining with sera from anti-CD45RC–treated *Aire^–/–^* animals, indicative of a decrease of circulating organ-specific autoantibodies in the preventive setting (Figure 2A, right). To further evaluate the levels of autoantibodies toward a larger panel of organs, we analyzed a spectrum of organs by Western blotting using sera from anti-CD45RC or isotype control mAb–treated *Aire^–/–^* animals on protein extracts from *IgM^–/–^* animals (Figure 2B). We observed numerous autoantibodies against multiple organ targets with the sera from isotype control mAb–treated *Aire^–/–^* animals (Figure 2B, top), and less reactivity with sera from anti-CD45RC mAb–treated *Aire^–/–^* animals (Figure 2B, bottom). These autoantibodies did not necessarily correlate with immune infiltrate in organs as previously described (18). Finally, we used the luciferase immunoprecipitation system (LIPS) assay to measure anti-cytokine autoantibodies, as well as antibodies specific to tissue-restricted antigens, i.e., protein disulfide isomerase–associated 2 (Pdia2), a pancreas-specific antigen expressed by medullary thymic epithelial cells in an AIRE-dependent manner (Figure 2C). These autoantibodies are present in *Aire^–/–^* animals, in particular against IFN-α11, IFN-α4, and IL-17A, and are undetectable in WT animals, as previously shown (18). We observed a trend toward decreased anti–IFN-α11, anti–IFN-α4, anti–IL-17A, and anti-rPdia2 autoantibody levels in rats treated with anti-CD45RC mAbs compared with isotype control mAbs (Figure 2C). We did not observe differences in other anti-cytokine autoantibodies analyzed such as anti–IL-22 and anti–IFN-α2, -α1, and -α7 (data not shown).

Taken together, these data indicate that anti-CD45RC mAb administration results in abrogated development of autoantibodies.

### Anti-CD45RC mAb leads to a specific depletion of CD45RC^hi^ Tconv cells with enrichment of CD45RC^lo/–^ Tregs in the preventive setting.

**Figure 3:**
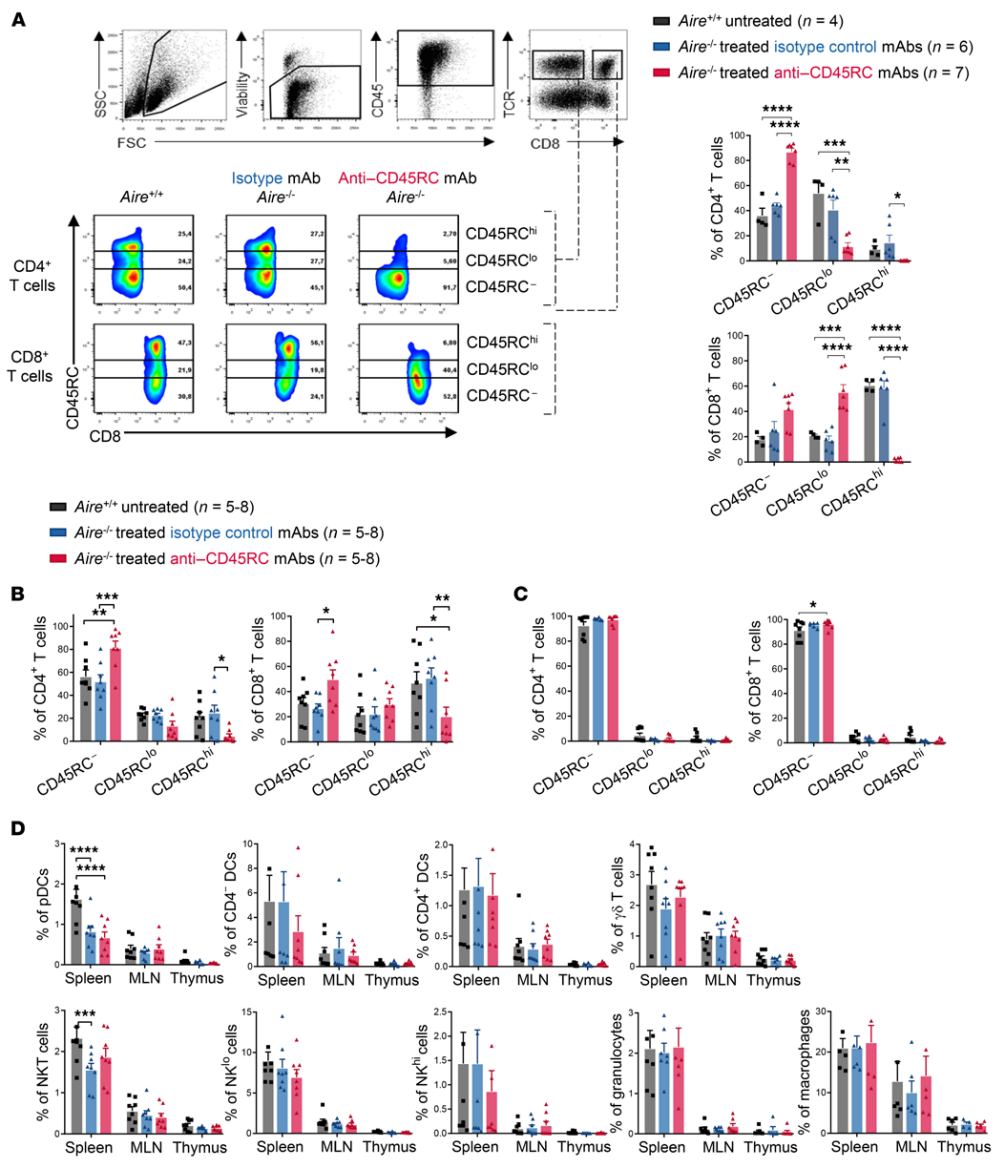
Anti-CD45RC mAb specifically depletes CD45RC^hi^ Tconv cells and increases CD45RC^lo/–^ Tregs. (**A**–**C**) Blood at 2 weeks of treatment (**A**) and spleen (**B**) and thymus (**C**) at 4 months of treatment of *Aire^–/–^* rats with isotype control or anti-CD45RC mAbs were stained for the expression of CD45RC on CD8^+^ and CD4^+^ T cells by flow cytometry and compared with those from *Aire*^+/+^ rats. (**A**) Shown is a representative staining of 4–7 animals. The gates indicate the high, low, and negative subsets of CD45RC. Mean ± SEM of CD45RC expression on CD4^+^ and CD8^+^ T cells after 2 weeks of treatment is summarized in the graphs on the right. ANOVA: *P < 0.05, **P < 0.01, ***P < 0.001, ****P < 0.0001. (**B** and **C**) Results are shown as mean ± SEM of CD45RC subsets among CD4^+^ T cells (left) or CD8^+^ T cells (right). ANOVA: *P < 0.05, **P < 0.01, ***P < 0.001. (**D**) Cell subset distribution was analyzed by flow cytometry among immune cells from spleen, mesenteric lymph nodes (MLN), and thymus of untreated *Aire^+/+^* and isotype control or anti-CD45RC mAb–treated *Aire^–/–^* rats at the time of sacrifice. Results are shown as mean ± SEM. ANOVA: ***P < 0.001, ****P < 0.0001.

To gain further mechanistic insight into how anti-CD45RC mAb ameliorates autoimmune lesions in *Aire*^–/–^ rats, we analyzed the efficacy of CD45RC^hi^ T cell depletion in this model. To detect CD45RC^+^ cells by flow cytometry, we used an anti-CD45RC mAb (clone 3H1437) that recognizes a different epitope from the OX22 clone used in vivo for the treatment, as previously described (23, 24). Analysis at day 14 after treatment initiation showed a significant decrease in both CD45RC^hi^ CD4^+^ and CD45RC^hi^ CD8^+^ T cells in anti-CD45RC mAb–treated *Aire^–/–^* animals compared with *Aire^–/–^* animals treated with the isotype control mAb or *Aire^+/+^* animals (Figure 3A). The effect of the anti-CD45RC mAb in decreasing CD45RC expression was greater in the blood on CD4^+^ T cells compared with CD8^+^ T cells, as we observed a stronger reduction of CD45RC^lo^ cells in this subset. *Aire* deficiency itself did not influence the expression of CD45RC (Figure 3A).

We confirmed the efficacy of the depletion at the time of animal sacrifice following 4 months of treatment in the spleen of anti-CD45RC mAb–treated *Aire^–/–^* animals for both CD4^+^ and CD8^+^ T cells, with an increase of regulatory CD45RC^lo/–^ T cells and a decrease of the pathogenic CD45RC^hi^ T subset (Figure 3B). We did not observe significant differences in the percentage of the CD45RC^lo^ subset, similarly to our previous observations in the transplantation and graft-versus-host disease models (23, 24). Analysis of the expression of CD45RC in the thymus demonstrated that while CD4^+^ and CD8^+^ T cells in the thymus of *Aire^+/+^* and *Aire^–/–^* rats were mainly CD45RC^–^, the proportion of CD45RC^–^ cells was slightly increased following the injection of the anti-CD45RC antibody (Figure 3C).

To further characterize the effects of the anti-CD45RC depleting mAb in vivo on immune cell subsets, we analyzed the proportion (Figure 3D) and absolute numbers (Supplemental Figure 1, B–E, and Supplemental Figure 2) of different immune cell populations based on their immunophenotype. We demonstrated previously that the CD45RC isoform is expressed by not only a fraction of T cells, but also other immune cells, such as B cells, NK cells, plasmacytoid dendritic cells (pDCs), and a fraction of NKT cells (23). We did not observe any differences in the percentage and absolute numbers of CD4^–^ and CD4^+^ DCs, γδ T cells, NK cells, macrophages, or granulocytes following anti-CD45RC mAb therapy in *Aire^–/–^* animals. We observed a significant reduction of pDCs and NKT cells in the spleens of *Aire^–/–^* versus *Aire^+/+^* WT animals, but this decrease was not significantly affected by the anti-CD45RC mAb treatment. In contrast, NKT cells were less affected by AIRE deficiency when animals were treated with the anti-CD45RC mAb. Taken together, these results indicate that anti-CD45RC mAb monotherapy reduces pathogenic Tconv cells throughout the duration of the mAb administration.

### Anti-CD45RC mAb acts directly on B cells by inhibiting their activation and secretion of immunoglobulins in vitro.

**Figure 4:**
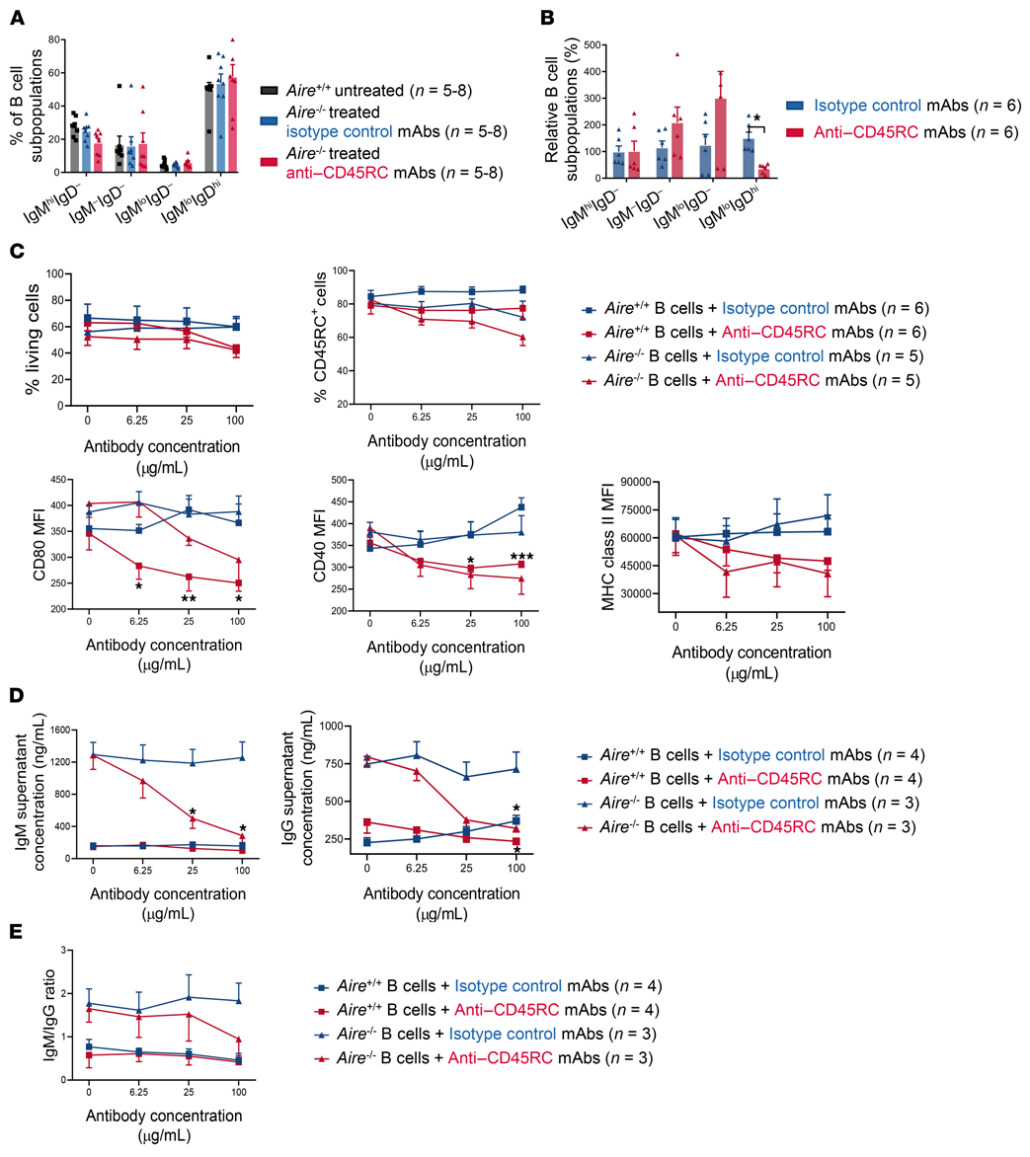
Anti-CD45RC mAb acts on B cells to decrease their activation and inhibit production of immunoglobulins in vitro. (**A**) Proportion of B cell subtypes in the spleen of *Aire^–/–^* rats after 4 months of treatment with the anti-CD45RC or isotype control mAb or WT untreated rats. (**B**) B cells from *Aire^+/+^* rats were stimulated in vitro for 48 hours with CpG ODN 1668, anti-CD40, and anti-IgM mAbs in the presence of anti-CD45RC or isotype control mAbs. Maturation of B cells was analyzed by flow cytometry quantification of each B cell subpopulation. ANOVA: *P < 0.05. (**C**) After 48 hours of in vitro stimulation, B cells from *Aire^+/+^* and *Aire^–/–^* rats were stained for flow cytometry to study their viability and expression of CD45RC and activation markers such as CD80, CD40, and MHC class II (RT1-B). Data from each experiment were normalized to the mean value from all the experiments. Results of multiple *t* test statistical analysis comparing isotype control with anti-CD45RC mAb conditions in *Aire^–/–^* or *Aire^+/+^* B cells. Multiple *t* test: *P < 0.05, **P < 0.01, ***P < 0.001. (**D**) IgM and IgG ELISA quantification in the culture supernatant of B cells stimulated for 48 hours. Multiple *t* test: *P < 0.05. (**E**) Ratio of IgM versus IgG production in culture supernatant of B cells stimulated for 48 hours.

To further decipher how anti-CD45RC mAb administration led to decreased autoantibodies, we analyzed B cell subpopulations in the spleen from *Aire^–/–^* animals at the end of the 4 months of treatment. We observed no significant difference in the percentage (Figure 4A) and absolute numbers (Supplemental Figure 1D) of each B cell subpopulation between *Aire^+/+^* and *Aire^–/–^* animals treated with either the isotype control or the anti-CD45RC mAb; these data are in agreement with previous findings that showed that the anti-CD45RC mAb has no depleting effects on B cells (23, 24). To gain more insights into the potential effects of the anti-CD45RC mAb on B cells, we stimulated B cells in vitro for 48 hours with TLR9 ligand (CpG ODN 1668) and anti-CD40 and anti-IgM antibodies in the presence of anti-CD45RC mAb. Flow cytometry analysis of surface immunoglobulin expression demonstrated that B cell stimulation in the presence of anti-CD45RC mAb significantly decreased the proportion of IgM^lo^IgD^hi^ B cells in comparison with isotype control mAb (Figure 4B), suggestive of an inhibitory effect on the maturation of naive B cells. The viability of B cells and the proportion of CD45RC^hi^ cells remained stable when cultured in the presence of anti-CD45RC or isotype control mAbs (Figure 4C), in accordance with our in vivo observations. Moreover, we found a dose-dependent decrease in the expression of the B cell activation markers MHC class II, CD40, and CD80 when cells were cultured in the presence of the anti-CD45RC mAb compared with the isotype control mAb. To elucidate whether the anti-CD45RC mAb affected isotype switching of B cells, we quantified the concentrations of IgM and IgG in the culture supernatant. We first demonstrated that after nonspecific stimulation in the absence of isotype control mAb or anti-CD45RC mAb, B cells from *Aire^–/–^* rats produced more IgM (6-fold) and IgG (3-fold) compared with B cells from *Aire^+/+^* rats. Interestingly, we observed that both IgM and IgG concentrations decreased to the level of *Aire^+/+^* B cells in the presence of anti-CD45RC mAb, at 25 μg/mL for IgG and 100 μg/mL for IgM (Figure 4D). Analysis of the IgM/IgG ratio demonstrated no significant differences in the presence of anti-CD45RC mAb (Figure 4E), suggesting that the anti-CD45RC mAb induces a global antibody reduction rather than inhibiting isotype switching. Collectively, these results suggest that anti-CD45RC mAbs can act directly on B cells by inhibiting their activation and blocking their immunoglobulin-secreting capacity. These mechanisms may be operational in vivo, via disruption of the B-T cell crosstalk, leading to the observed decrease of autoantibody production in *Aire^–/–^* treated rats.

### Anti-CD45RC mAb switches the Treg/Tconv balance and restores the altered transcriptomic profile of CD45RC^lo/–^ Tregs in Aire-deficient rats.

**Figure 5:**
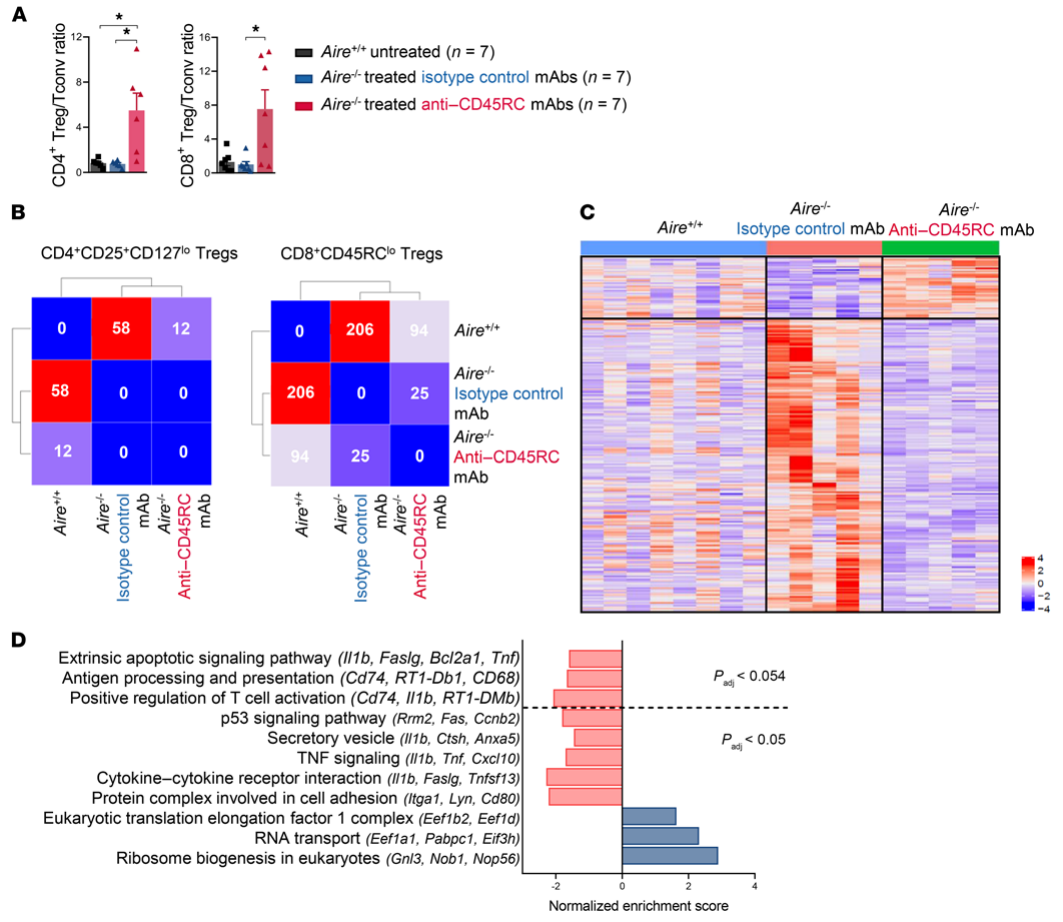
Anti-CD45RC treatment switches the Treg/Tconv balance and restores the altered transcriptomic profile of Tregs in *Aire^–/–^* rats. (**A**) Ratio of CD4^+^CD25^+^CD127^lo/–^ or CD8^+^CD45RC^lo/–^ Tregs versus CD45RC^hi^ Tconv cells in *Aire^–/–^* rats treated with isotype control (*n =* 7) versus anti-CD45RC mAb (*n =* 7). ANOVA: *P < 0.05. (**B**) Matrix showing the number of genes differentially expressed between CD4^+^ (left panel) and CD8^+^ (right panel) Tregs from the following groups: WT rats (*n =* 8) and *Aire^–/–^* rats treated with the isotype control (*n =* 5) or the anti-CD45RC mAb (*n =* 5). (**C**) DGE RNA sequencing heatmap analysis of CD8^+^CD45RC^lo^ Tregs showing the relative expression of genes. Columns correspond to samples, and rows correspond to differentially expressed genes. Expression values were averaged per sample and scaled per gene. Blue represents lowly-expressed genes and red represents highly expressed genes. (**D**) Normalized enrichment score of biological pathways upregulated or downregulated in *Aire^–/–^* rats treated with the anti-CD45RC mAb (*n =* 5) compared with *Aire^–/–^* rats treated with the isotype control mAb (*n =* 5).

APECED patients and *Aire^–/–^* mice exhibit a defect in Tregs’ suppressive activity (26, 27). In a rat model of allogeneic solid organ transplantation, we previously demonstrated that short-term treatment with anti-CD45RC mAb increased the number and improved the function of CD4^+^ and CD8^+^ Tregs (23). We first assessed the CD4^+^ and CD8^+^ Treg/Tconv cell ratio and observed a significant change in the ratio in favor of CD4^+^CD25^+^CD127^lo/–^ and CD8^+^CD45RC^lo/–^ Tregs in anti-CD45RC mAb–treated *Aire^–/–^* animals (Figure 5A). Then, to characterize the in vivo effects of anti-CD45RC mAb on Tregs in the context of AIRE deficiency and further define the biological consequences of its administration, we analyzed differential gene expression upon RNA sequencing of FACS-isolated CD45RC^lo/–^ CD8^+^ Tregs and CD25^hi^CD127^lo/–^ CD4^+^ Tregs from *Aire^+/+^* animals and *Aire^–/–^* animals treated with either the isotype control or the anti-CD45RC mAb (Figure 5, B–D). We found that the transcriptomic signatures of both CD4^+^ and CD8^+^ Tregs of anti-CD45RC–treated *Aire^–/–^* rats were more similar to the transcriptomic signatures of *Aire^+/+^* rats than to those of *Aire^–/–^* rats treated with isotype control mAb (Figure 5B), showing that the anti-CD45RC mAb administration induced a reversal toward a normal expression of some of the genes downregulated or upregulated in *Aire^–/–^* Tregs. This gene signature reversal trend was especially striking in the CD8^+^ Tregs, with 206 genes being differentially expressed between *Aire^+/+^* and *Aire^–/–^* isotype control–treated rats against only 94 genes differentially expressed between *Aire^+/+^* and *Aire^–/–^* anti-CD45RC–treated rats (Figure 5C). Significantly upregulated genes after anti-CD45RC treatment belonged to pathways involved in translation and ribosome machinery (e.g., *Rpl6*, *Rpl5*, *Rpl7*, *Eef1a1*), while genes significantly downregulated belonged to pathways involved in apoptosis (*Fas*, *Ccnb2*, *Rrm2*), cell adhesion (*Itga1*), and inflammatory conditions (*Il1b*, *Ccl1*, *Ccr6*, *Cxcl10*, *Pdcd1*, *Faslg*, *Tnfsf13*, *Tnf*) (Figure 5D). Taken together, these results demonstrate that in vivo administration of anti-CD45RC mAb reverses the Treg transcriptomic gene signature of *Aire^–/–^* animals toward that of *Aire^+/+^* animals, consistent with the effect on Tconv cell depletion and the ensuing decrease in the inflammatory microenvironment.

### Anti-CD45RC mAb immunotherapy in Aire^–/–^ rats more than 3 months old inhibits the development of autoimmune symptoms.

**Figure 6:**
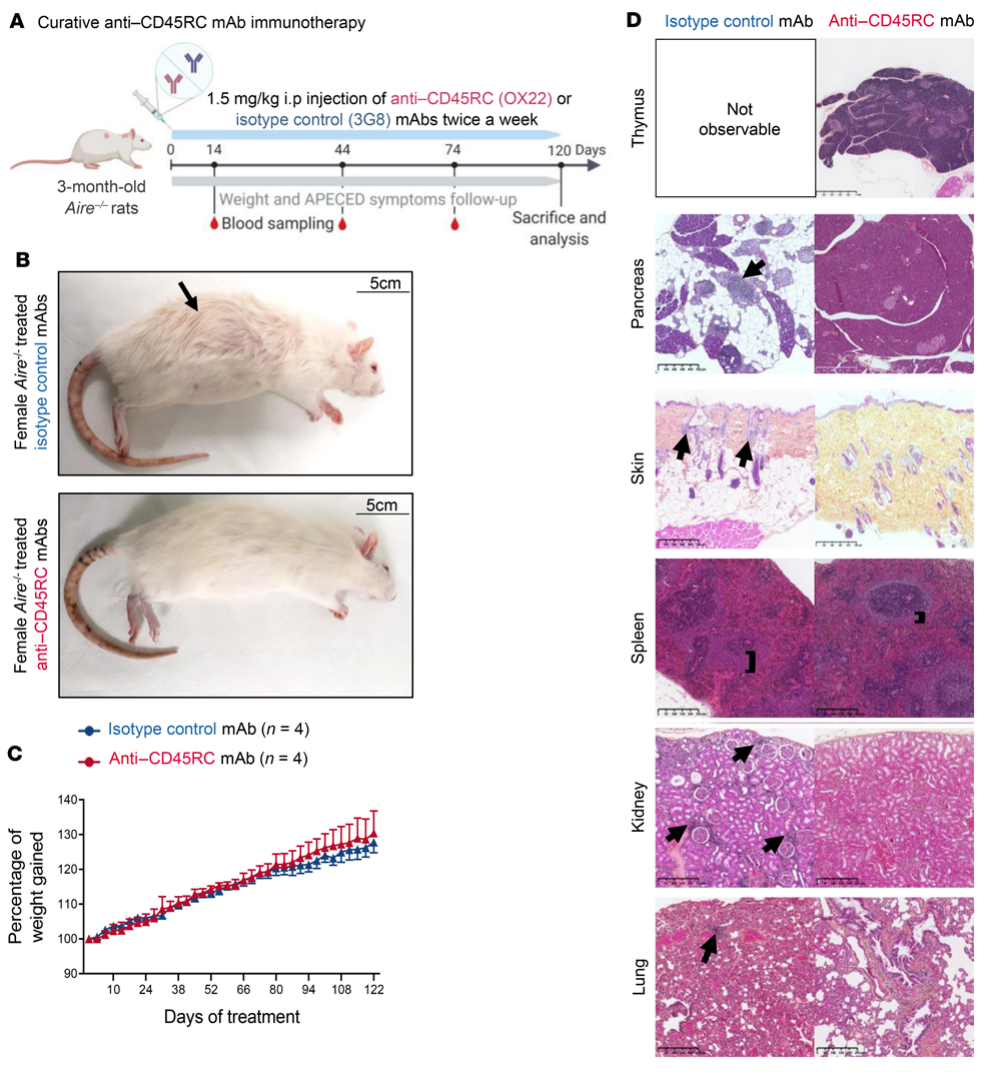
Anti-CD45RC mAb administration initiated as treatment controls the development of autoimmune lesions in *Aire^–/–^* rats. (**A**) Schematic of the protocol of treatment in the curative setting of 3-month-old *Aire^–/–^* rats with isotype control or anti-CD45RC mAbs. (**B**) Representative photographs of *Aire^–/–^* rats after 4 months of treatment with either isotype control (*n =* 8) or anti-CD45RC mAbs (*n =* 11). Arrows depict alopecia. Scale bars: 5 cm. (**C**) Evolution of weight gain in male *Aire^–/–^* rats treated with isotype control (*n =* 4) or anti-CD45RC mAbs (*n =* 5) until sacrifice. ANOVA analysis showed no significant difference between the 2 groups. (**D**) Representative pictures of H&E histological analysis of organs from *Aire^–/–^* rats at the end of the treatment with isotype control (*n =* 4) or anti-CD45RC mAbs (*n =* 5). Black arrows indicate autoimmune lesions and immune cell infiltrates. Black bars indicate the marginal zone. Scale bars: 250 nm (spleen, lung, and kidney), 500 μm (skin and pancreas), and 1 mm (thymus).

**Figure 7:**
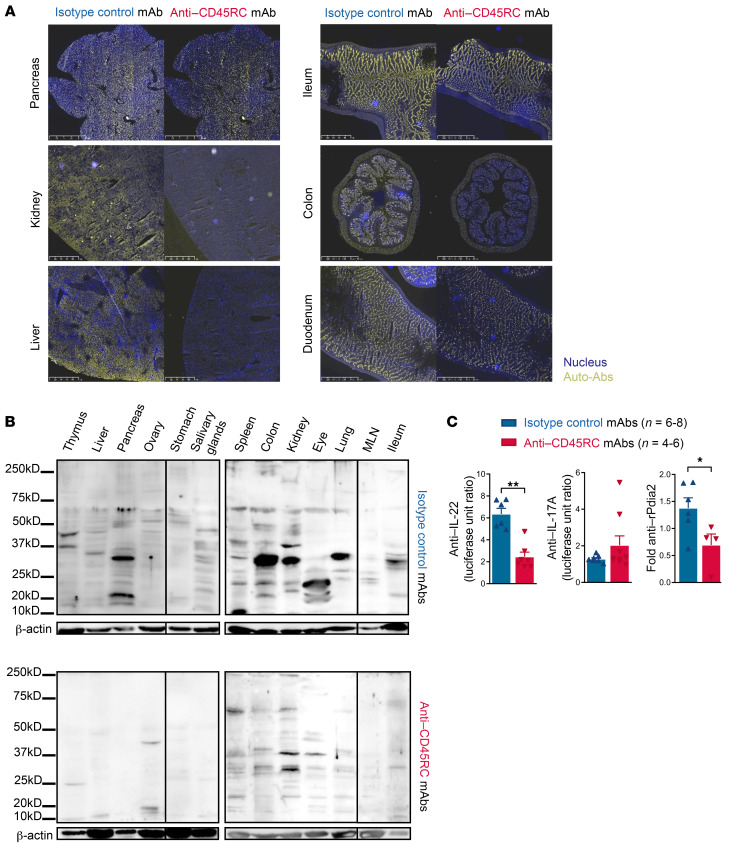
Anti-CD45RC mAb immunotherapy initiated as treatment decreases the production of autoantibodies in *Aire^–/–^* rats. (**A**) Organ serial sections from the same *IgM^–/–^* rats as shown in Figure 2 were incubated with different sera from anti-CD45RC or isotype mAb–treated *Aire^–/–^* animals at 4 months of treatment as indicated. Autoantibodies are depicted in yellow and DAPI in blue. Original magnification, ×20. Images are representative of 3 animals per group. (**B**) Sera from anti-CD45RC or isotype mAb–treated *Aire^–/–^* rats at 4 months of treatment were incubated on Western blot membranes after migration and transfer of tissue-specific self-antigens from *IgM*^–/–^ rats. β-Actin was used as a loading control. Data are representative of 5 animals per group. MLN, mesenteric lymph nodes. (**C**) Anti-cytokine and -Pdia2 autoantibodies were quantified by LIPS assay using sera from anti-CD45RC or isotype mAb–treated *Aire^–/–^* rats at 4 months of treatment (*n =* 5 each). Normalization was achieved by division of the obtained value by the mean values obtained from WT animals. Mann-Whitney: *P < 0.05, **P < 0.01.

Since the majority of APECED patients are only diagnosed and treated after the first symptoms, we investigated the efficacy of the anti-CD45RC mAb in a treatment rather than a preventive setting. To that end, anti-CD45RC (*n =* 11) or isotype control (*n =* 8) mAbs were given twice weekly in 3-month-old *Aire^–/–^* animals just before appearance of the first visible disease sign, corresponding to the time when the weight curve starts to plateau. Treatment was continued for 4 months until animal sacrifice (Figure 6A). None of the animals had been sacrificed before the end of the experimental setup. Using this approach, we observed that the anti-CD45RC mAb inhibited the development of visible manifestations in *Aire^–/–^* animals compared with isotype control mAb–treated *Aire^–/–^* animals as evidenced by the absence of alopecia- and vitiligo-like symptoms (Figure 6B). However, we did not observe any impact of the immunotherapy on weight growth (Figure 6C), probably because the growth of the animals had reached a plateau at that age range in contrast to Figure 1C. In contrast, we observed a marked improvement in the histological appearance of organs in anti-CD45RC mAb–treated compared with isotype control mAb–treated *Aire^–/–^* animals (Figure 6D and Table 1), as shown by the preserved exocrine pancreas structure, the slight reduction of spleen follicle marginal zone, and the decreased cell infiltration in the kidney and lung. These observations were associated with decreased autoantibody production as shown by immunofluorescence staining (Figure 7A), Western blotting (Figure 7B), and anti–IL-22 and anti-rPdia2 assessed by LIPS assay (Figure 7C).

### CD45RC expression is increased in peripheral blood T cells from APECED patients and reflects the immune system imbalance induced by AIRE deficiency.

**Figure 8:**
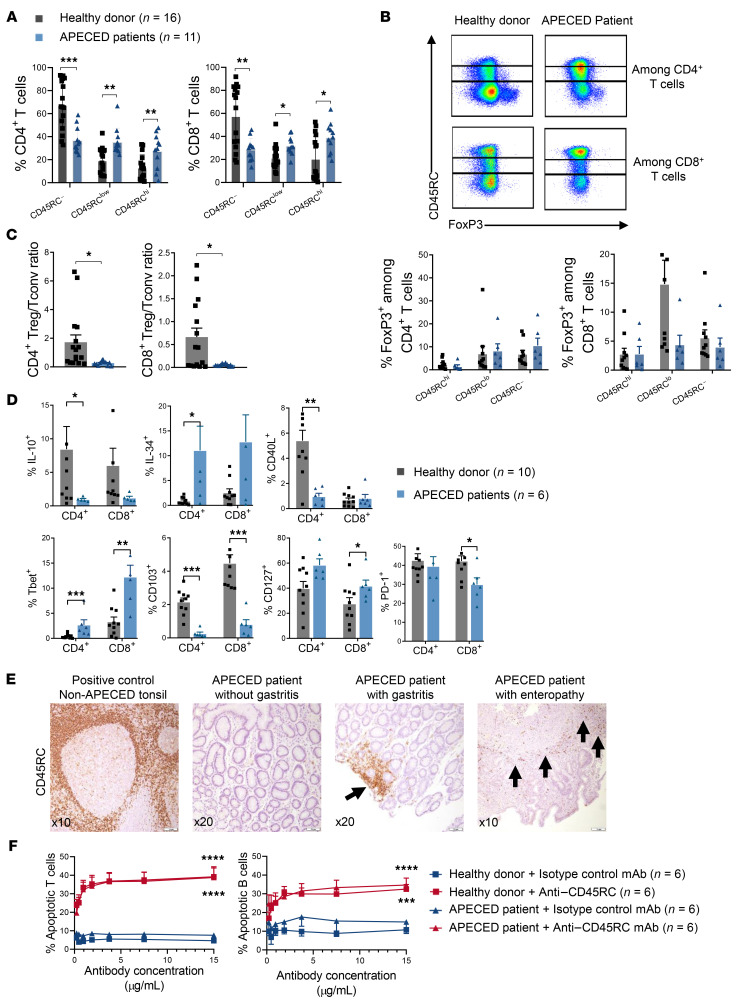
CD45RC expression is increased in peripheral blood T cells and autoimmune tissue lesions of APECED patients. (**A**) PBMCs of APECED patients (*n =* 11) and healthy donors (*n =* 16) were stained for flow cytometry analysis showing the expression of CD45RC on CD4^+^ (left) and CD8^+^ (right) T cells; *t* test: *P < 0.05, **P < 0.01, ***P < 0.001. (**B**) Expression of FOXP3 and CD45RC on CD4^+^ (top line) and CD8^+^ T cells (bottom line) from APECED patients and healthy donors. (**C**) Ratio of FOXP3^+^ Tregs versus CD45RC^hi^ Tconv cells in healthy donors (*n =* 16) versus APECED patients (*n =* 11); *t* test: *P < 0.05. (**D**) Expression of IL-10, IL-34, CD40L, Tbet, CD103, CD127, and PD-1 by CD4^+^ and CD8^+^ T cells from healthy donors and APECED patients; *t* test: *P < 0.05, **P < 0.01, ***P < 0.001. (**E**) Representative immunohistochemical staining of CD45RC with an anti–human CD45RC mAb in stomach and small intestine paraffin-embedded tissue from 2 APECED patients with autoimmune gastritis and enteropathy (arrows) compared with stomach tissue of an APECED patient without autoimmune gastritis. Non-APECED human tonsil biopsy tissue was used as positive control. (**F**) Proportion of apoptotic CD45RA^hi^ T cells induced after a 3-hour in vitro incubation of PBMCs, from healthy donors (*n =* 6) or APECED patients (*n =* 6) with the anti-CD45RC or isotype control mAbs. One-way ANOVA repeated measures, Bonferroni’s post hoc test: ***P < 0.001, ****P < 0.0001.

We performed a preliminary evaluation for the potential of targeting CD45RC in APECED patients, by looking for the expression of CD45RC in blood and tissue specimens of APECED patients. Flow cytometry analysis of PBMCs from 11 APECED patients revealed that CD45RC was significantly upregulated on CD4^+^ and CD8^+^ T cells compared with healthy individuals (Figure 8A), with a significantly greater proportion of CD45RC^hi^ and CD45RC^lo^ cells and a significantly lower proportion of CD45RC^–^ cells. This decrease in CD45RC^–^ cells correlated with a decrease in the percentage of CD8^+^FOXP3^+^ T cells, since FOXP3^+^ cells are included mostly within the CD45RC^lo/–^ populations (Figure 8B and ref. 23), and a significant imbalance of the ratio of FOXP3^+^ Tregs versus CD45RC^hi^ CD4^+^ and CD8^+^ Tconv cells (Figure 8C). On the functional side, we observed that T cells from APECED patients produced less IL-10 (both CD4^+^ and CD8^+^), PD-1 (CD8^+^), CD40L (CD4^+^), and CD103 (CD4^+^ and CD8^+^) and expressed more IL-34 (CD4^+^ and CD8^+^), Tbet (CD4^+^ and CD8^+^), and CD127 (CD8^+^) (Figure 8D).

To investigate whether lymphocytic infiltrates in organs affected by autoimmunity in APECED patients were composed of CD45RC^+^ cells, we performed immunostaining of CD45RC on tissue biopsies from 6 patients. We first confirmed that CD45RC^+^ cells could be detected by immunostaining on paraffin biopsies in non-APECED human tonsil tissue (Figure 8E). Interestingly, numerous CD45RC^+^ cells were present in the stomach (*n =* 3) and small intestine (*n =* 1) of APECED patients with gastritis or enteropathy, respectively, whereas no CD45RC staining was observed in patients without autoimmune gastritis (*n =* 2).

Finally, we validated the efficacy in vitro of an anti–human CD45RC mAb (clone ABIS-45RC, human IgG1) to induce cell death of target CD45RC^hi^ T cells in PBMCs from APECED patients (*n =* 6) similarly to PBMCs from healthy individuals (Figure 8F, left). Analysis of the effect of the anti–human CD45RC mAb on CD45RC^hi^ B cells revealed a similar depleting effect in PBMCs from APECED patients (*n =* 11) compared with healthy individuals (*n =* 16) (Figure 8F, right), suggesting that the human IgG1 isotype used induced potent effector functions resulting in not only inhibition but elimination of peripheral CD45RC^hi^ B cells.

Taken together, these results indicate that dysregulated and inflammatory CD45RC^hi^ cells circulate in blood and infiltrate organs affected by autoimmunity and could be targeted by therapeutic intervention with a specific mAb against CD45RC.

## Discussion

In this article we report the potential of an immunotherapeutic approach using anti-CD45RC mAb to treat the autoimmune syndrome APECED. Targeted immunotherapies have opened a new era of opportunities to treat rare and multiorgan autoimmune diseases that are more challenging to treat than more frequent autoimmune diseases affecting wider patient populations. Typically, the study of rare diseases can be difficult because of the lack of animal models, the paucity of patients, and the difficulty of executing powered clinical trials (17). Although APECED is a rare disease, its prevalence can reach higher proportions in some populations, such as 1:25,000 in Finns and 1:9000 in Iranian Jews (28, 29), and the recent description of dominant monoallelic mutations associated with a later onset of the disease and a milder phenotype suggests that disease-causing AIRE mutation is underestimated (30). The clinical therapeutic options for APECED patients are limited to a combination of symptomatic nonspecific treatments adapted to each patient that are not only inefficient in the long term, but even deleterious in many ways (6, 31–33). In this study we took advantage of a previously generated rat model of AIRE deficiency mimicking some of the key features of APECED syndrome, which are not found in mouse *Aire^–/–^* models, to evaluate the effects of targeted therapy (18). The only reported biological used in immunotherapy of APECED is the anti-CD20 mAb rituximab, used with relative success to treat pneumonitis in APECED patients in combination with T cell–targeted immunomodulators (19, 34). T cell–targeted immunotherapy alone or even nonablative hematopoietic stem cell transplantation, which has proven to be efficient in immune dysregulation polyendocrinopathy enteropathy X-linked (IPEX) syndrome, another primary immune deficiency disorder (35), has never been tested in APECED to the best of our knowledge. Besides having fewer side effects, targeted immunotherapies have the advantage that their target and their efficacy on target-expressing cells can additionally be validated ex vivo before administration to patients, as we have shown here on the blood cells and tissues of APECED patients for the anti-CD45RC mAb. CD45RC could even serve as a biomarker for precision medicine of patient stratification and follow-up.

We compared effectiveness using 2 different interventional strategies, i.e., as prevention and treatment, applicable to APECED patients, who are often diagnosed clinically when they present at least 2 symptoms from the Whitaker triad before the age of 20 years. Using these 2 interventional strategies, we demonstrate in both cases that 4 months of exposure to anti-CD45RC mAb immunotherapy efficiently controls disease onset and halts progression in the long term in our animal model of APECED. The anti-CD45RC mAb treatment was more efficient in the preventive than in the curative setting, in which anti-CD45RC mAb could only halt progression, and in which, although visual manifestations are not visible yet, organ lesions may already be present and, in some organs, irreversible. One example is the involution of the thymic structure that occurs earlier in AIRE-KO rats and is probably due to the dysregulation of the thymopoiesis and autoimmune responses toward the thymus (18). Although the anti-CD45RC mAb treatment does not resurrect the thymus, it might prevent immune responses toward the thymus and those involved in thymic destruction. It would be interesting to compare thymus involution over time in anti-CD45RC mAb–treated AIRE-KO rats versus WT. Although APECED is a genetic disease due to the loss of AIRE, a central tolerance regulator, the disease’s components involve T and B cells responsible for tissue damage (3, 36–38). This allowed us to speculate that specific targeting of CD45RC, a splicing variant of the CD45 molecule, which is expressed by, e.g., naive precursors of Tconv cells , Tconv cells, and effector memory T cells, but also by B cells, is a reasonable approach in the treatment of APECED patients before too much tissue damage has taken place (22, 23, 39). This protein is a transmembrane tyrosine phosphatase that acts as an essential regulator of T and B cell antigen receptor signaling in the immunological synapse by tuning the activity of Lck in T cells or Lyn, Fyn, and Lck in B cells (40–42). We previously demonstrated that short-term targeting of CD45RC using an mAb that induces T cell death is an efficient strategy to ameliorate solid organ transplant rejection (23) and graft-versus-host disease (24), and that long-term treatment was efficient in preventing muscle strength loss in Duchenne’s muscular dystrophy, a monogenic disease with CD45RC^+^ T cell inflammatory infiltration in muscle (25). In the APECED model, by directly targeting the autoreactive T cells expressing CD45RC, the anti-CD45RC mAb controlled the T cell–dependent tissue cytotoxicity, but also, by controlling T-B cell crosstalk and directly inhibiting B cells, anti-CD45RC mAb can additionally prevent the production of autoantibodies (3), a feature that was not previously evidenced with this antibody (23–25). Indeed, although B cells are mostly CD45RC^hi^, we previously demonstrated that the anti–rat CD45RC mAb induced apoptosis of T cells, but not B cells, through signaling and activation of the intrinsic apoptosis pathway involving mitochondria (23). We also showed that the mechanism of action of the anti-CD45RC mAb did not rely on antibody-dependent cell-mediated cytotoxicity through recruitment of effector cells or complement-dependent cytotoxicity. To further discriminate a potential impact of the anti-CD45RC mAb on B cells through modulation of isotype switching or maturation, we further analyzed the impact of the antibody on B cells in vitro. We first demonstrated that after nonspecific stimulation, B cells from *Aire^–/–^* rats produce more immunoglobulins compared with B cells from *Aire^+/+^* rats, something that was not demonstrated with our model yet and that suggests that AIRE deficiency results in the modification of B cell function. In addition, for the first time to our knowledge, we showed that although B cells are not depleted, the anti-CD45RC mAb controls immunoglobulin secretion and prevents, to some extent, B cell activation. B cells seem to contribute to APECED disease, as demonstrated by B cell–deficient *Aire^–/–^* mice that had ameliorated multiorgan infiltration (36), by B cell depletion in NOD *Aire^–/–^* mice that led to diabetes onset delay (43), and by NOD *Aire*^–/–^*Ighm*^–/–^ mice that had partial amelioration of autoimmune lung disease (19). Interestingly, *Aire^–/–^* rats display splenic abnormalities with a hypertrophic marginal zone, already described in *Aire^–/–^* mice (44), suggesting chronic antigen exposure and enhanced immune responses. The marginal zone is mainly composed of B cells that mostly recognize antigens in a T cell–independent manner (45). While these observations do not resolve the link between AIRE and B cell autoimmunity, the fact that *Aire^–/–^* rats that received anti-CD45RC mAb have a normal marginal zone is consistent with our in vitro observations, since it suggests that this effect could be mediated by the direct binding of the anti-CD45RC mAb on B cells and their consecutive inhibition. In addition, the presence of anti-CD45RC mAb bound to CD45RC^+^ B cells could also directly interfere with the MHC-TCR interaction. Finally, by restoring the transcriptomic profile of Tregs and increasing Treg numbers, thus rebalancing the ratio of Tregs to Tconv cells involved in the peripheral selection of B cells (46–48), anti-CD45RC mAb restores central checkpoints for B cell selection. Thus, the anti-CD45RC mAb mechanism of action involves B cell inhibition in addition to CD45RC^hi^ T cell depletion and Treg activation. Still, the role of B cells and autoantibodies in the context of APECED syndrome needs to be investigated further, and the *Aire^–/–^* rat model, which develops anti-cytokine autoantibodies at levels comparable to those in APECED patients, will be useful in future studies, including those involving the generation of double AIRE- and immunoglobulin-deficient rats, which is under way.

Importantly, our results support the potential of anti-CD45RC mAb in a preventive setting that is applicable to familial aggregations of APECED syndrome. Although our strategy does not correct the *Aire* gene deficiency, it efficiently prevents the autoimmune process. In addition, current gene editing technologies do not allow for effective corrective gene therapy in the context of APECED syndrome. A recent study with *Aire^–/–^* mice demonstrated an adeno-associated virus (AAV) approach to correct AIRE deficiency, which was able to significantly reduce the development of autoimmune symptoms; however, this therapeutic effect was limited by technical issues linked to medullary thymic epithelial cells (mTECs) and AAV biology (49). First, the lack of mTEC-specific AAV serotype leads to the need of a direct injection inside the thymus and results only in a proportion of AIRE^+^ mTECs. Second, mTECs are a rare cell population with a rapid turnover, and thus the use of a non-integrative transgene, although safer, might not allow for stable expression and long-lasting therapeutic effects. Therefore, before an efficient *Aire* editing strategy is reached, immunotherapy seems the best asset to prevent multiorgan tissue damage and to treat patients affected by APECED. Interestingly, we observed that CD45RC^hi^ cells were not only present but also increased in APECED patients despite their ongoing treatments (most patients receive endocrine hormone replacement therapy, including physiological levels of fludrocortisone and hydrocortisone); thus we can speculate that the steroids received will not interrupt anti-CD45RC mAb treatment.

Finally, the APECED animal model could serve as a disease model for many serious autoimmune diseases involving T and B cell dysregulation, such as rheumatoid arthritis or systemic lupus erythematosus, and it is reasonable to speculate that our observations of effectiveness of anti-CD45RC mAb in APECED could be extended to other autoimmune diseases with similar imbalance.

In summary, we show that anti-CD45RC mAb can eliminate autoreactive T cells and control/restore B cell and Treg tolerance in the AIRE-deficient rat model of APECED disease. The anti-CD45RC mAb immunotherapy was effective both as prevention and as treatment, the latter being particularly relevant to the human disease. We also show that AIRE deficiency in APECED patients disrupts the immune balance toward an autoimmune and inflammatory state with increased levels of CD45RC^hi^ in peripheral blood T cells and in autoimmune lesion lymphocytic infiltrates, highlighting the potential translational implications of anti-CD45RC immunotherapy in this disease.

## Methods

### Animals.

*Aire^–/–^* Sprague-Dawley (SPD) rats were obtained by backcrossing of the *Aire^–/–^* Brown Norway strain, generated using zinc finger nuclease technology as previously described (18), in the SPD background for more than 8 generations. The SPD animals used for this project were male and female and were all bred in our facility; WT animals were littermates of the *Aire^–/–^* rats. IgM-deficient rats used for Western blot and immunofluorescent staining were previously described (50). All studies were performed according to protocols approved by the Ethics Committee of the Ministère de l’Enseignement Supérieur et de la Recherche (Paris, France).

### Genotyping.

Small ear biopsies were taken from 10-day-old rats to perform genotyping as previously described (51). Briefly, samples were digested overnight at 56°C in 350 μL of lysis buffer, then diluted at 1:20 in ultrapure water and added to 24 μL of PCR mix reaction prepared according to the manufacturer protocol (Herculase II Fusion DNA Polymerase, Agilent Technologies). The following primers were used to carry out the PCR amplification: forward primer 5′-TCAAGAGTGCCCTGTTCTAG-3′, reverse primer 5′-CTGGGGTGGTGTCAGTAAG-3′. The amplification program was run on a Veriti Thermal Cycler (Applied Biosystems) and consisted of 1 cycle at 95°C for 5 minutes, 35 cycles at 98°C for 10 seconds, 60°C for 10 seconds, and 72°C for 30 seconds, followed by 1 cycle at 72°C for 4 minutes. Finally, to determine the presence of mutations in the *Aire* gene, the PCR product’s length was estimated using a heteroduplex mobility assay with a microfluidic capillary electrophoresis system (Caliper LabChip GX, PerkinElmer).

### In vivo antibody treatments.

Three-week-old (prevention) or 3-month-old (treatment) *Aire^–/–^* rats or WT littermates received 1.5 mg/kg purified mAbs, either isotype control or anti-CD45RC (respectively, clone 3G8, a mouse IgG1 against human CD16, or clone OX22, a mouse IgG1 against rat CD45RC; both produced in our laboratory from hybridoma culture), twice a week by i.p. injection. Treatments lasted 4 months; animals were daily surveyed and scored for the occurrence of APECED-like signs such as alopecia, vitiligo, nail dystrophy, and weight loss. At the end of the treatment, rats were sacrificed.

### Histological analysis.

Organs from *Aire^–/–^* treated rats were collected in 4% paraformaldehyde. After 1 week of fixation at 4°C, samples were embedded in paraffin, cut in sections of 0.2 μm, and stained with hematoxylin-eosin-saffron by the MicroPicell platform (SFR Bonamy). Images were captured using the Nanozoomer slide scanner (Hamamatsu). Lymphocyte infiltration and pathological changes in the tissues were assessed by a pathologist in a blinded manner.

### Cell isolation.

Spleens were perfused with collagenase D and dissociated before incubation at 37°C for 30 minutes; 350 μL of EDTA 0.01 mM was added to stop the reaction. Pieces of tissue were digested to generate single-cell suspensions before red blood cell lysis was performed using a hypertonic buffer (8.29 g NH_4_Cl, 1 g KHCO_3_, 37.2 mg EDTA per liter of sterilized water). Cells from thymus and mesenteric lymph nodes were mechanically dissociated.

### Antibodies and flow cytometry.

Before cell surface staining, 500,000 cells per sample were stained with 50 μL of diluted viability dye (Fixable Viability Dye eFluor 506, eBioscience) for 30 minutes at 4°C in the dark. After a washing step, extracellular staining was performed by incubation of the cells with 50 μL of diluted fluorescent antibodies (Supplemental Tables 1 and 2) for 30 minutes at 4°C in the dark. If intracellular staining was also performed, cells were next permeabilized and fixed for 30 minutes according to the protocol of the kit’s manufacturer (Foxp3/Transcription Factor Staining Buffer Set, eBioscience). Fluorescent antibodies targeting intracellular markers were diluted in the permeabilization buffer and incubated with the fixed cells for 1 hour. For cell phenotyping, fluorescence was measured with a BD FACSCanto II or an LSR II flow cytometer; a BD FACSAria was used for cell sorting (BD Biosciences). FlowJo software was used to analyze data after elimination of doublets and Viability^+^ dead cells.

Rat immune cell populations were defined as: CD4^+^CD25^–^ effector T cells (Tconv cells), CD4^+^CD25^+^CD127^lo/–^ and CD8^+^CD45RC^lo/–^ regulatory T cells (Tregs), TCRαβ^–^CD45RA^+^ B cells, TCRαβ^–^CD45RA^–^CD45R^+^CD4^+^ plasmacytoid dendritic cells (pDCs), TCRαβ^–^CD103^+^CD4^–^ and TCRαβ^–^CD103^+^CD4^+^ conventional DCs , CD11b/c^+^His48^+^ granulocytes, CD11b/c^+^His48^–^ macrophages, TCRγδ^+^ γδ T cells, TCRαβ^–^CD161^lo^ natural killer–low (NK^lo^) cells, TCRαβ^–^CD161^hi^ NK^hi^ cells, TCRαβ^+^CD161^+^ NKT cells.

### In vitro stimulation of B cells.

Splenocytes were mechanically dissociated from the spleen before lysing of red blood cells. After a washing step, cells were stained for 30 minutes with purified mouse anti-rat antibodies: anti-T (TCRαβ clone R7/3), macrophages and DCs (anti-CD11b/c clone OX42), Tγδ (anti-TCRγδ clone V65), and NK cells (anti-CD161 clone 3.2.3). Magnetic anti-mouse beads (Dynabead Goat Anti-Mouse IgG, Invitrogen) were used to deplete αβ and γδ T cells, DCs, and NK cells from the cell suspension. Fc receptor was then blocked by incubation in 10% heat-inactivated rat serum for 30 minutes. Cells were then washed and resuspended in complete RPMI 1640 medium before plating of 200,000 enriched B cells per well. B cells were stimulated in vitro with 5 μg/mL of coated anti-CD40 antibody (clone HM40-3) and 5 μg/mL of CpG ODN 1668 (InvivoGen); maturation of naive B cells was induced by coating of 10 μg/mL anti-IgM antibody (clone MARM-4). Increasing concentrations of anti-CD45RC antibody (clone OX22) or isotype control (clone 3G8) were added to each well. After 12, 24, and 48 hours of culture, viability and activation markers were analyzed by flow cytometry.

### Western blot.

Protein extracts from organs (skin, thymus, liver, pancreas, ovary, duodenum, stomach, salivary glands, spleen, colon, kidney, eye, lung, testis, mesenteric lymph nodes, ileum) of IgM-deficient rats (used to avoid background signals from immunoglobulins normally present in tissues) were obtained using a RIPA buffer and a gentleMACS dissociator (Miltenyi Biotec). After quantification with a bicinchoninic acid (BCA) assay (Thermo Fisher Scientific), 50 μg of protein from each organ were denatured by addition of DTT and heated at 95°C for 5 minutes. Samples were then run in a 10% Tris-SDS acrylamide gel and transferred onto a Protran premium 0.45 NC (0.45 μm) Western membrane (Amersham). Saturation was performed by incubation in TBST/5% BSA 5 solution for 2 hours. Next, membranes were incubated overnight at 4°C under constant agitation with sera from *Aire^–/–^* treated rats diluted at 1:200 in TBST/1% BSA. After washing with TBST, membranes were incubated with a secondary donkey anti-rat IgG antibody (heavy and light chain; Jackson ImmunoResearch Laboratories). After 2 hours of incubation under constant agitation at room temperature, autoantibodies were revealed by addition of SuperSignal West Pico chemiluminescent substrate (Thermo Fisher Scientific). Pictures were taken with an LAS 4000 gel imager (Fujifilm Life Science).

### Immunofluorescence and confocal microscopy.

Slides of organs (pancreas, kidney, liver, ileum, colon, and duodenum) from IgM-deficient rats were generated as mentioned before and frozen at –20°C until use. Just after thawing, slides were saturated with PBS plus 5% BSA for 30 minutes at room temperature. Serum from treated *Aire^–/–^* rats was diluted at 1:50 in PBS/1% BSA and added on the slices. After 30 minutes of incubation at room temperature, slices were washed 3 times with filtered PBS. Autoantibodies were revealed using an Alexa Fluor 488–coupled goat anti-rat IgG antibody (heavy and light chain; Invitrogen). After 30 minutes of incubation with the secondary antibody and another washing step, slices were stained with 2 μg/mL of DAPI for 15 minutes and then mounted with ProLong Gold Antifade reagent (Molecular Probes). Forty-eight hours later, the fluorescence was observed using the Nanozoomer slide scanner (Hamamatsu).

### ELISA quantification of rat immunoglobulins.

Plates were coated overnight at 4°C with 4 μg/mL of goat polyclonal anti-rat IgM or IgG antibodies (supplied by Jackson ImmunoResearch Laboratories). After several washing steps, saturation with PBS/5% BSA was performed for 2 hours at room temperature. Diluted samples and isotype control (rat IgM, IgG1, IgG2a, and IgG2b supplied by BD Biosciences), used to determine the standard curves, were incubated for 90 minutes at 37°C. HRP-conjugated anti-rat IgM, IgG1, IgG2a, or IgG2b (supplied by Bethyl Laboratories) was incubated for 90 minutes at 37°C to allow specific detection of rat immunoglobulin isotypes. Tetramethylbenzidine (TMB; BD Biosciences) was used as HRP chromogenic substrate before the reaction was stopped by addition of H_2_SO_4_. Optical density at 450 nm was determined using a Tecan Spark plate reader.

### Luciferase immunoprecipitation system.

Anti-cytokine autoantibodies were detected using a luciferase immunoprecipitation system (LIPS) assay on a 96-well MultiScreen filter HTS plate (Millipore) as previously described (52). Briefly, HEK293 cells were transfected with plasmids coding for rodent antigens and with the NanoLuc gene to secrete NanoLuc-cytokine fusion proteins. Culture supernatants were incubated overnight at 4°C with diluted rat serum (1:10 or 1:25) and with the antigen-NanoLuc fusion proteins (containing 1 × 10^6^ luciferase units). Immune complexes formed were captured onto protein G agarose beads (25 μL of 4% suspension; Exalpha Biologicals). One hour later, the plate was washed and the NanoLuc substrate (furimazine, Promega) was added. The intensity of luminescence was recorded for 5 minutes by a VICTOR X5 plate reader (PerkinElmer).

### 3′ Digital gene expression RNA sequencing.

3′ Digital gene expression (3′DGE) RNA sequencing was performed as previously described in detail (23). RNA was extracted from FACS-isolated Tregs using the RNeasy Micro Kit (QIAGEN) according to the supplier protocol. Libraries were prepared with 10 ng of total RNA. During template switching of reverse transcriptase, the poly(A) tail of mRNA was tagged with universal adapters, barcodes, and unique molecular identifiers (UMIs). All the cDNAs were then pooled, amplified, and tagged using the transposon-fragmentation approach. This library of 350–800 bp was run on an Illumina HiSeq.

The read pairs were used for the analysis only if they reached quality controls: For read 1, the first 6 bases must correspond exactly to the barcode sequence and the next 10 bases correspond to UMIs. Alignment of the second read to the RefSeq rat mRNA sequences (rn6) was done using Burrows-Wheeler Aligner version 0.7.4 with non-default parameter –1,24. Reads were excluded from the analysis if they mapped to multiple positions of the genome. We obtained the DGE profiles by counting the number of UMIs associated with each RefSeq gene for each sample.

Differentially expressed genes between conditions were calculated using the R package DESeq2 (Bioconductor) by first applying a regularized log transformation (rlog). Genes with adjusted *P* value inferior to 0.05 were considered as differentially expressed. Heatmaps were generated by scaling and centering gene expression (gene expression was transformed so that the mean = 0 and standard deviation = 1). For functional enrichment analysis, databases (Kyoto Encyclopedia of Genes and Genomes [KEGG], Reactome, and Gene Ontology) and the R package fgsea were used to identify significantly enriched or depleted groups of genes in each condition. The raw DGE RNA sequencing data are available in the European Nucleotide Archive (ENA) (https://www.ebi.ac.uk/ena/browser/home, accession number PRJEB43753).

### APECED patient and healthy donor blood samples.

APECED patients were followed at the Inflammation Center, Helsinki University Hospital, or at the National Institute of Allergy and Infectious Diseases at the NIH, Bethesda, Maryland, USA, and gave written informed consent to PBMC donation.

All patients were under endocrine hormone replacement therapy, including physiological levels of fludrocortisone and hydrocortisone for adrenal insufficiency. One patient was on continuous cyclosporine therapy due to kidney transplantation.

Blood from healthy donors was collected at the Etablissement Français du Sang. PBMCs were purified from blood by Ficoll-Paque density gradient centrifugation (Eurobio, Courtaboeuf) before freezing in FCS/10% DMSO. Samples were preserved at –150°C before use.

### APECED patient biopsies and immunohistochemistry.

Stomach (*n =* 5) and small intestine (*n =* 1) biopsies obtained from 6 APECED patients followed at the National Institute of Allergy and Infectious Diseases at the NIH, Bethesda, Maryland, USA, were analyzed in this study. The patients were enrolled on an NIH IRB–approved protocol and signed written informed consent for study participation in accordance with the Declaration of Helsinki. Immunohistochemical stains were performed on formalin-fixed, paraffin-embedded tissue. Slides were deparaffinized in xylene (3 times for 5 minutes) and rehydrated in graded alcohol (4 times for 2 minutes) and distilled water. Heat-induced antigen retrieval was performed using high-pH buffer in a steamer for 30 minutes. The slides were immersed in 3% Tris–goat serum solution to block nonspecific binding, and then 1:200 CD45RC (clone MT2, catalog AM39022PU-N, Origene) antibody was applied at room temperature and incubated for 1 hour. Detection was performed on a Benchmark Ultra automated immunostainer (Roche) with an ultraView Universal DAB Detection Kit (catalog 760-500). The slides were then dehydrated in graded alcohols followed by xylene treatment (twice for 5 minutes) and coverslipped.

### Anti-CD45RC mAb–induced apoptosis on APECED patients’ PBMCs.

At day –1, PBMCs from APECED patients (*n =* 6) and healthy donors (*n =* 6) were thawed and incubated at 37°C, 5% CO_2_, in complete RPMI 1640 medium to allow recovery. After overnight incubation, cells were harvested and centrifuged to discard dead cells. Fifty thousand cells were then seeded in duplicate in a 96-well round-bottom plate before addition of increasing concentrations of either the anti-CD45RC mAb (clone ABIS-45RC, AbolerIS Pharma) or an isotype control mAb (human IgG1, anti–β-gal, InvivoGen). After 3 hours of incubation at 37°C, 5% CO_2_, cells were harvested, washed, and stained for 30 minutes at 4°C with fluorescent anti-CD3, anti-CD19, and anti-CD45RA mAbs. Apoptosis was assessed by annexin V staining. Cells were washed once with PBS before being incubated for 20 minutes at room temperature with 25 μL of annexin V–APC (BD Biosciences) diluted in annexin V binding buffer (BD Pharmingen). Without washing, 75 μL of DAPI was added to each well, and results were immediately read in FACSCanto (BD Biosciences). The mean percentage of annexin V^+^CD45RA^hi^ T or B cells in negative control, consisting of cells incubated without any antibody, was withdrawn from each condition to only quantify the antibody-induced apoptosis.

### Statistics.

We used 2-way ANOVA, 2-tailed *t* test, and multiple *t* test statistical analysis in FACS, and Mann-Whitney test for ELISA and LIPS experiments. A *P* value of less than 0.05 was considered significant.

### Study approval.

Experiments and procedures were performed in accordance with protocols approved by the Ethics Committee of the Ministère de l’Enseignement Supérieur et de la Recherche (Paris, France; APAFIS22646).

The study was approved by the Ethics Committee of Medicine, Helsinki and Uusimaa Joint Authority (HUS/1127/2016). The patients were enrolled on an NIH IRB–approved protocol (11-I-0187) and signed written informed consent for study participation in accordance with the Declaration of Helsinki.

## Author contributions

CG conceived, designed, and funded the study and interpreted data. CG and MB wrote the manuscript. MB, CS, JO, AM, NV, LF, HS, GS, SP, EMNF, and CU acquired, analyzed, and interpreted data. IA, AR, MSL, and PP provided essential reagents and interpreted data. The manuscript was edited by all authors.

## Supplementary Material

Supplemental data

## Figures and Tables

**Table 1:**
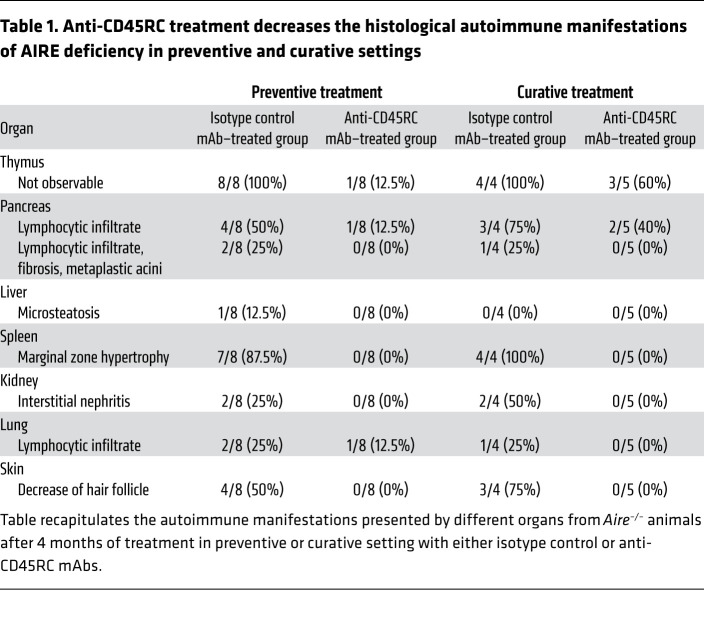
Anti-CD45RC treatment decreases the histological autoimmune manifestations of AIRE deficiency in preventive and curative settings

## Version 1 02/15/2022

In-Press Preview

## Version 2 04/01/2022

Electronic publication
