# Supplementary material for: Anti-CD45RC antibody immunotherapy prevents and treats experimental autoimmune polyendocrinopathy–candidiasis–ectodermal dystrophy syndrome
Source: J Clin Invest. 2022 Apr 1;132(7):e156507. doi: 10.1172/JCI156507 (PMC8970675; doi:10.1172/JCI156507)
Supplement: Supplemental data [file jci-132-156507-s022.pdf]

Suppl. Figure 1. Besnard et al.

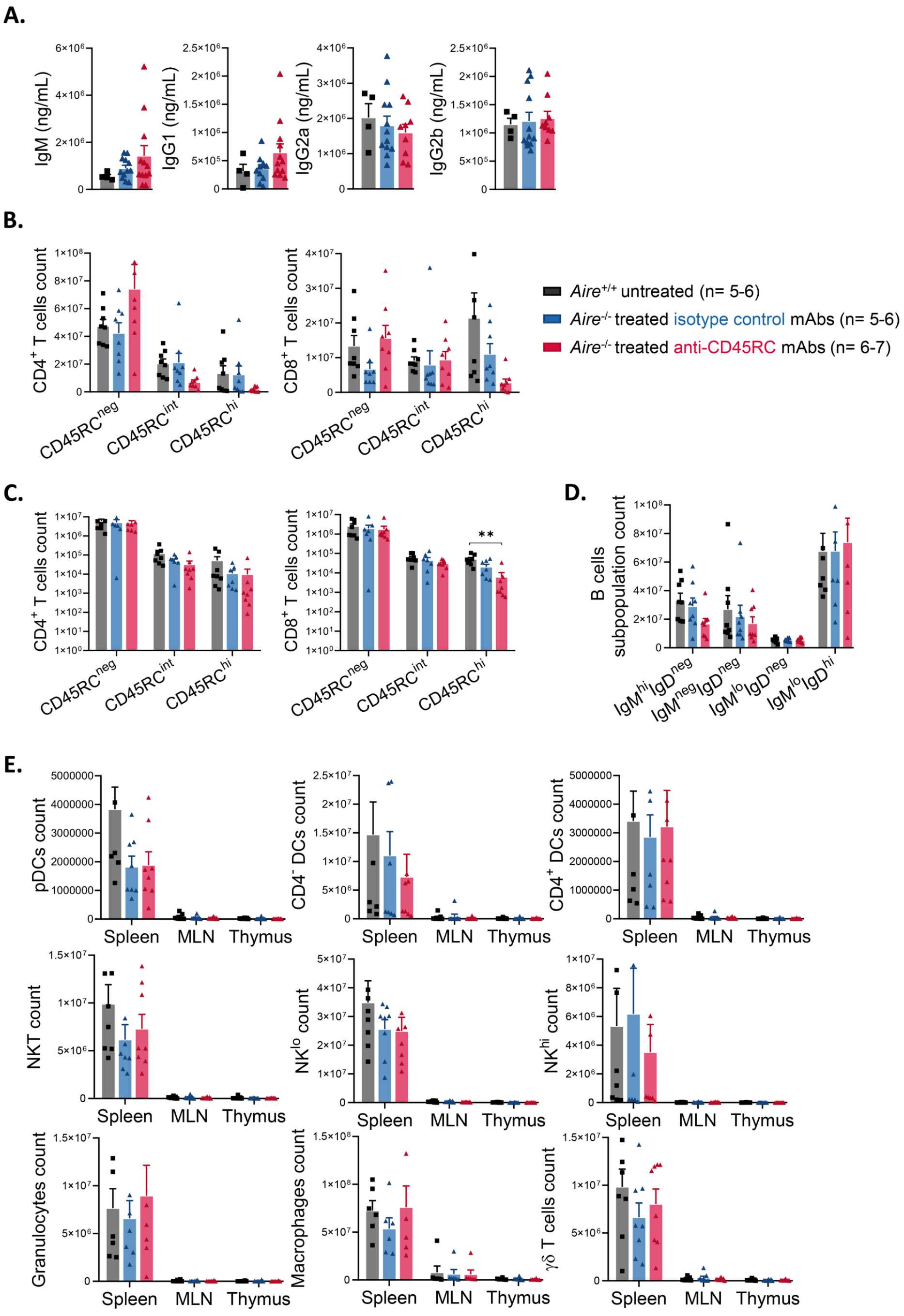

Suppl. Figure 1: Anti-CD45RC mAbs specifically depletes CD45RC$^+$ effector T cells. (A) IgM and IgG subtypes 1, 2a and 2b concentration were assessed by ELISA in sera from isotype control or anti-CD45RC mAbs-treated *Aire*$^{-/-}$ rats compared to *Aire*$^{+/+}$ animals taken at the end of the four-month period of treatment. Results are shown as mean ± SEM. CD45RC expression on

$CD4^+$ (left) and $CD8^+$ (right) T cells were analyzed on splenocytes (**B**) and thymocytes (**C**) from *Aire*$^{+/+}$ and *Aire*$^{-/-}$ rats after 4 months of treatment with the isotype control or anti-CD45RC mAbs. **(D)** B cells subpopulations in the spleen of *Aire*$^{+/+}$ and *Aire*$^{-/-}$ rats at the end of the treatment. **(E)** Phenotyping by flow cytometry of immune cells from spleen, MLN and thymus of *Aire*$^{+/+}$ and *Aire*$^{-/-}$ treated rats. **, $p<0.01$.

Suppl. Figure 2. Besnard et al.

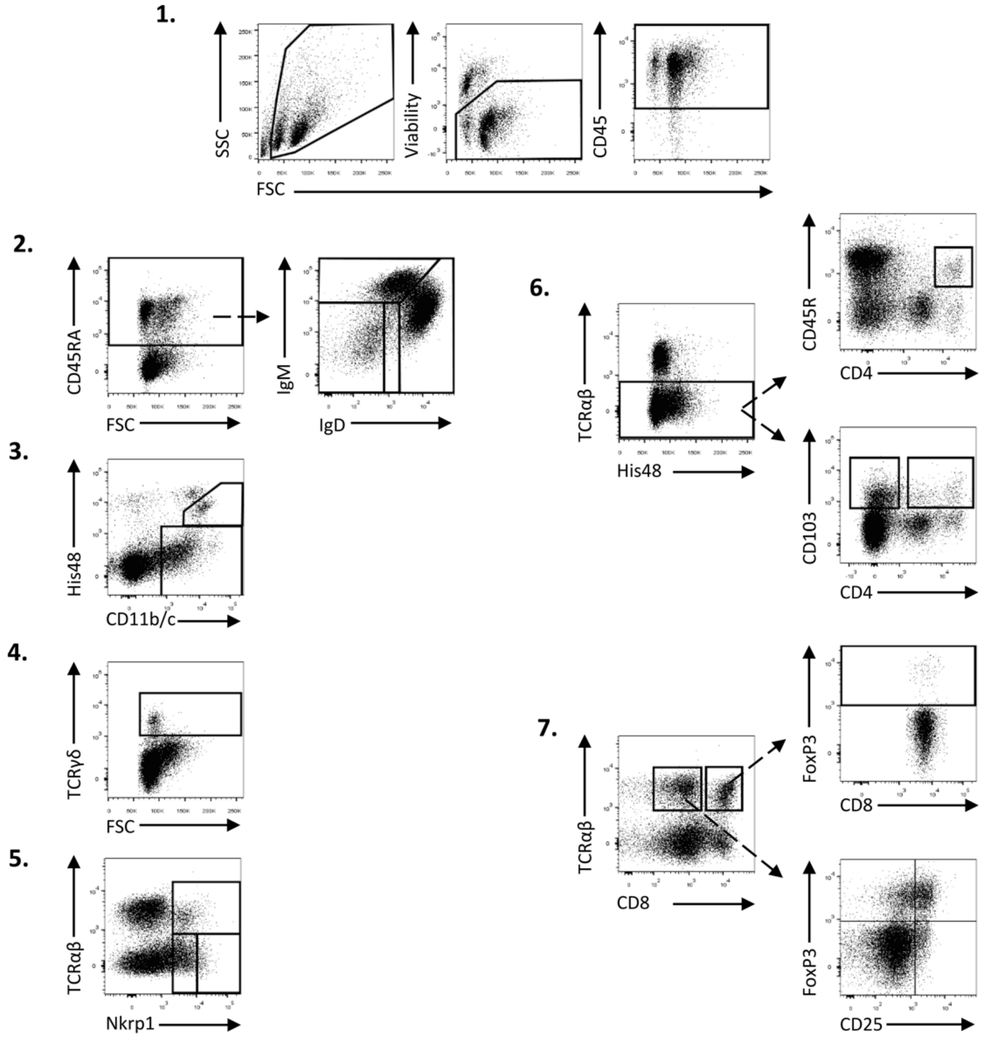

Suppl. Figure 2: Gating strategy for immune cell phenotyping. (1) Dot plot of the first gates common to each cell populations. Specific gating strategies for (2) B cell subpopulations, (3) granulocytes and macrophages, (4) γδT cells, (5) NK, (6) dendritic cells and (7) Treg populations.

Suppl. Table I. Antibody used to define rat immune cells

Marker	Clone	Provider
CD4	OX35	BD Biosciences
CD8	OX8	In-house
CD25	OX39	In-house
CD40	HM 40-3	BD Biosciences
CD45	OX1-OX30	BD Biosciences
CD45R	His24	BD Biosciences
CD45RA	OX33	In-house
CD45RC	OX22	In-house
	3H1437	SantaCruz
CD80	3H5	BD Biosciences
CD103	OX62	In-house
CD127	Polyclonal	Thermofisher
CD161	3.2.3	In-house
85C7	85C7 FX	In-house
FoxP3	Fkj-16s	Thermofisher
IgM	MARM-4	Biorad
IgD	MARD	Biorad
TCRab	R7/3	BD Biosciences
TCRgd	V65	In-house

Suppl. Table II. Antibody used to stain human cells

Marker	Clone	Provider
CD3	UCHT1	BD Biosciences
CD4	SK3	BD Biosciences
CD27	M-T271	BD Biosciences
CD28	CD28.2	BD Biosciences
CD45RA	HI100	BD Biosciences
CD45RC	MT2	IQ-products
CD103	Ber-ACT8	BD Biosciences
CD127	hIL-7R-M21	BD Biosciences
CD154	TRAP1	BD Biosciences
CD279	EH12.1	BD Biosciences
FoxP3	259D/C7	BD Biosciences
GITR	REA841	Miltenyi
HLA-DR	L243	BD Biosciences
IFNg	B27	BD Biosciences
IL-10	JES3-9D7	BD Biosciences
IL-34	IC5265P	R&D Systems
Tbet	O4-46	BD Biosciences
TGFb1	TW4-9E7	BD Biosciences
